# Supplementary material for: A Comprehensive Clinical Outcome Analysis of Endoscopic Vessel Harvesting for Coronary Artery Bypass Surgery
Source: J Clin Med. 2024 Jun 11;13(12):3405. doi: 10.3390/jcm13123405 (PMC11204017; doi:10.3390/jcm13123405)
Supplement: Supplementary file 1 [file jcm-13-03405-s001.zip › jcm-3034288-supplementary.pdf]

Supplementary Table S1: Overall clinical outcomes of all CABG cases under this study

**Outcome variable (n=1884)**	**Frequency**	**Percent**
Renal complication (AKI)	41	2.2
Renal complications (Renal failure)	12	0.6
Cause of mortality (Cardiac)	12	0.6
Cause of mortality (Neurologic)	4	0.2
Cause of mortality (Renal)	2	0.1
Cause of mortality (Vascular)	1	0.1
Cause of mortality (Infection)	4	0.2
Cause of mortality (Pulmonary)	3	0.2
Cause of mortality (Other)	7	0.4
Perioperative MI	0	0
Radial artery	84	4.5
Saphenous vein	1387	73.6
Number of distal coronary anastomoses		
1	28	1.5
2	319	16.9
3	1071	56.8
4	428	22.7
5	37	2.0
6	1	0.1
Use of cardiopulmonary bypass (CPB)		
Full	1830	97.1
None	50	2.7
Combined	3	0.2
Converted	1	0.1
Cannulation methods		
Aorta and atrial/caval	1802	95.6
Femoral artery and femoral/jugular	27	1.4
Aorta and femoral/jugular vein	3	0.2
Femoral artery and atrial/caval	2	0.1
Aortic occlusion		
Cross-clamp	1360	72.2
Balloon	1	0.1
Intracoronary shunt used	236	12.5
Intra aortic balloon pump (IABP) used		
Pre-operative	216	11.5
Intra-operative	155	8.2
Post-operative	12	0.6

Supplementary Table S2: Clinical outcomes of the EVH procedure

Outcome variable (n=1418)	Frequency	Percent
Type of system used (SVG)		
- Marquet	1333	70.8
- Terumo	52	2.8
Location of incision (SVG)		
- Both legs	84	4.5
- Left leg	1215	64.5
- Right leg	86	4.6
Length of incision (SVG) EVH only		
- 2.5cm	1360	72.2
- Others	23	1.2
Use of CO2 (SVG) - Yes	1362	72.3
Quality of vessel (SVG) (choice=Good quality and calibre)	1202	63.8
Quality of vessel (SVG) (choice=Varicosities)	17	0.9
Quality of vessel (SVG) (choice=Sclerotic)	8	0.4
Quality of vessel (SVG) (choice=Small calibre)	118	6.3
Quality of vessel (SVG) (choice=Dense adhesions)	446	23.7
Quality of vessel (SVG) (choice=Many tributaries)	483	25.6
Quality of vessel (SVG) (choice=Others)	89	4.7
Conversion of indication from endoscopic to open	10	0.5
Total number of incisions for harvest (SVG)		
- 1 incision	1300	69.0
- 2 incision	76	4.0

Supplementary Table S3: Multivariate regression analysis of leg-wound complications

Variables in the Equation	P Value	OR 95% C.I. (Lower-Upper)
**Leg wound complications**		
Method of harvest	<.001	1.946 (1.528 - 2.477)
Gender	0.051	0.740 (0.547 - 1.002)
Diabetes	0.722	0.959 (0.764 - 1.206)
Renal Disease	0.075	1.421 (0.965 - 2.093)
Left Main Stem Disease	0.143	1.196 (0.941 - 1.520)
EF	0.405	0.896 (0.692 - 1.160)
Logistic Euroscore	0.034	1.020 (1.001 - 1.039)
**Leg wound infection**		
Method of harvest	0.050	1.517 (0.999 - 2.303)
Diabetes	0.005	1.837 (1.205 - 2.800)
Renal Disease	0.565	1.198 (0.647 - 2.220)
Peripheral vascular disease	0.031	1.793 (1.055 - 3.048)
Left Main Stem Disease	0.135	1.362 (0.908 - 2.042)
EF	0.294	1.258 (0.819 - 1.931)
Logistic Euroscore	0.257	1.016 (0.989 - 1.044)
**Leg wound hematoma**		
Method of harvest	0.039	0.402 (0.169 - 0.957)
Diabetes	0.290	0.732 (0.410 - 1.306)
Hyperlipidemia	0.065	1.871 (0.961 - 3.642)
Left Main Stem Disease	0.506	1.220 (0.679 - 2.189)
EF	0.130	0.312 (0.069 - 1.410)
Logistic Euroscore	0.965	0.999 (0.941 - 1.060)

OR= Odds ratio; EF= Ejection Fraction
